# Changes in central venous pressure during a fluid challenge have limited value for guiding fluid therapy

**DOI:** 10.62675/2965-2774.20240073-en

**Published:** 2024-11-14

**Authors:** Priscilla Souza de Oliveira, Fernando José da Silva Ramos, Daniere Yurie Vieira Tomotani, Flávia Ribeiro Machado, Flávio Geraldo Rezende de Freitas

**Affiliations:** 1 Universidade Federal de São Paulo Escola Paulista de Medicina Department of Anesthesiology, Pain and Intensive Care Medicine São Paulo SP Brazil Department of Anesthesiology, Pain and Intensive Care Medicine, Escola Paulista de Medicina, Universidade Federal de São Paulo - São Paulo (SP), Brazil.

**Keywords:** Central venous pressure, Critical care, Fluid therapy, Respiration, artificial, Cardiac output

## Abstract

**Objective:**

To evaluate whether changes in central venous pressure during fluid expansion and baseline cyclic respiratory variation in the central venous pressure amplitude (RespCVP) curve could be used to discriminate between fluid responders and nonresponders.

**Methods:**

This prospective observational study included critically ill adult patients who underwent fluid expansion in the form of a fluid bolus or fluid challenge with crystalloids. All patients were under mechanical ventilation and adequately sedated. We determined the central venous pressure at baseline (CVPT0) and the changes at 5 (ΔCVPT5), 10 (ΔCVPT10) and 15 (ΔCVPT15) minutes during fluid infusion. We also measured the RespCVP at baseline. Fluid responsiveness was defined as a cardiac index increase of ≥ 15%.

**Results:**

The study included 30 patients (11 responders and 19 nonresponders). The CVPT0 and the changes after a fluid challenge at all three time points did not adequately predict fluid responsiveness, as determined by their area under the curve values (CVPT0: 0.70, (95%CI: 0.49 - 0.90; ΔCVPT5: 0.78, (95%CI: 0.57 - 0.99; ΔCVPT10: 0.63, (95%CI: 0.39 - 0.88; ΔCVPT15: 0.68, ((95%CI: 0.45 - 0.92). The RespCVP at baseline also had a poor performance (area under the curve: 0.70; 95%CI: 0.50 - 0.91).

**Conclusion:**

Changes in central venous pressure have limited value in predicting fluid responsiveness.

## INTRODUCTION

Intravenous fluids are generally regarded as the first step in the resuscitation of critically ill patients with signs of poor tissue perfusion.^(1,2)^ The rationale for fluid administration is to increase venous return, which leads to an increase in the cardiac output and improved organ perfusion.^(3)^ However, clinical studies in heterogeneous populations of critically ill patients have demonstrated that the cardiac output increases in only approximately 50% of hemodynamically unstable patients after fluid infusion.^(4,5)^

Central venous pressure (CVP) is one of the most traditional hemodynamic variables used to predict fluid responsiveness. Its widespread use is based on the dogma that CVP reflects the intravascular volume.^(6)^ However, CVP represents a complex interplay of factors, including cardiac compliance, right ventricular function, intrathoracic pressure, the vascular tone, and the circulating volume. Consequently, changes in CVP may not solely reflect alterations in the cardiac preload. Numerous studies have convincingly demonstrated that absolute values of CVP do not accurately reflect the intravascular volume and are ineffective in distinguishing responders from nonresponders.^(2,3,6-11)^ Despite long-standing evidence suggesting that CVP is not adequate for guiding fluid therapy, a large observational study was published in 2015, which described how physicians conduct fluid challenges in intensive care and revealed that CVP was still the preferred variable for predicting fluid responsiveness.^(12)^

Unlike static CVP values, there is a general belief that dynamic changes in CVP could be useful, especially when more advanced monitoring systems are not available.^(13-15)^ The most commonly used strategy is based on the variations in CVP in the course of a fluid challenge to guide subsequent fluid infusions, as proposed by Weil and Henning in the "5-2" rule.^(16)^ According to this strategy, minimal changes in CVP, such as an increase below 2 mmHg, may be indicative of fluid responsiveness, leading to additional administration of fluids.^(13,16,17)^ Another interesting method is the cyclic variation in the amplitude of CVP (RespCVP) in patients under mechanical ventilation, as proposed by Westphal et al.^(18)^ This index was previously reported to be in good agreement with pulse pressure variation, suggesting that it may be used as a dynamic marker of fluid responsiveness.

Thus, we hypothesized that changes in CVP during fluid expansion in critically ill patients under mechanical ventilation could help identify the fluid responsiveness status. We also aimed to assess whether the baseline RespCVP could accurately predict fluid responsiveness.

## METHODS

This prospective observational study was carried out in two mixed intensive care units (35-bed and 16-bed) in two teaching hospitals. The study was approved by the ethical committees of both institutions. Deferred informed consent was obtained from the patients’ legal representatives.

We included adult patients who received fluid expansion in the form of a fluid bolus or fluid challenge with crystalloids (either Ringer's lactate or sodium chloride as a 0.9% solution, with 500mL infused over 15 minutes), as indicated at the discretion of the attending physician. Only patients receiving continuous sedation, fully adapted to the ventilator, and monitored with an arterial catheter and a device to measure the cardiac output were eligible for inclusion in this study. We excluded patients with active bleeding (suspected or confirmed), an urgent need for fluid replacement, arrhythmias, known intracardiac shunts, tricuspid disease, and poor-quality CVP tracing.

We used a multiparameter bedside monitor (DX 2020, Philips-Dixtal, São Paulo, Brazil) for pressure measurements. We determined the CVP at baseline and at 5, 10 and 15 minutes of fluid infusion. All CVP measurements were performed in triplicate and averaged. We measured the CVP through a central venous catheter in all the patients, including those who were monitored with a pulmonary artery catheter. We determined the position of the central venous catheter tip through a bedside chest radiograph. To determine the CVP, we froze the monitor screen with simultaneous CVP tracing, electrocardiogram, and capnography signals. We positioned the cursor line at the base of the "a" wave at the end of expiration.^(19)^ Changes in CVP during a fluid challenge were defined as follows: ΔCVPT5, ΔCVPT10, and ΔCVPT15 were the differences between the CVP values measured at 5, 10 and 15 minutes and at baseline, respectively. The amplitude of the CVP was calculated from the top to the bottom part of the "a" wave tracing, both at the inspiratory phase (CVPins) and at the expiratory phase (CVPexp). We calculated the RespCVP according to the following formula: ΔCVP(%)=[( CVPexp− CVPins)/ CVPexp]×100.^(18)^

At baseline and immediately after the fluid challenge, a set of hemodynamic variables, including the heart rate, mean arterial pressure, and cardiac index, were recorded. To ensure the accuracy of intravascular pressure measurements, we carefully observed the patency of the line, the leveling of the transducer (midaxillary line at the fourth interspace), the zeroing at atmospheric pressure, and the quality of the signal (square wave testing). All other treatments, including sedative and vasoactive drugs, were not changed during the study.

We measured the cardiac index via a PiCCO system (Pulsion Medical Systems SE, Munich, Germany), a VolumeView/EV1000 system (Edwards Lifesciences, Irvine, CA, USA) or a semicontinuous cardiac output with a pulmonary artery catheter (Vigilance, Edwards Lifesciences, Irvine, CA, USA). For the PiCCO and EV1000 systems, the cardiac index was determined via pulse contour analysis. According to the local protocol, the calibration was repeated every eight hours or after a major change in the patient's clinical condition. For the pulmonary artery catheter, we considered the average value of five consecutive measurements from the STAT mode screen of the Vigilance® monitor (Edwards Lifesciences, Irvine, CA, USA) following completion of the fluid challenge. Patients were classified as responders if the fluid challenge induced an increase in the cardiac index of ≥ 15%.

We also obtained clinical and epidemiological variables through medical records. We calculated the Sequential Organ Failure Assessment (SOFA) score on the day of the protocol and the Simplified Acute Physiology Score (SAPS) 3 on admission to the intensive care unit (ICU).

### Statistical analysis

To calculate the sample size, we considered an area under the receiver operating characteristic (ROC) curve of 0.85 or higher for the variable ΔCVPT15 to predict fluid responsiveness. Using an alpha level of 0.05, a beta level of 0.20, and a ratio of responders to nonresponders of 1:1, a minimum of 9 patients per group was needed.

The data are expressed as the numbers (percentages), means ± standard deviations, or medians (interquartile ranges), as appropriate. Continuous data were tested for normality of distribution via the Shapiro–Wilk test. To compare the CVP values between responders and nonresponders over time, we used a mixed model with individual random effects. We tested the correlation between changes in the cardiac index (in percentages) and changes in CVP following a fluid challenge (ΔCVPT15) via the Spearman rank method.

Receiver operating characteristic curves were constructed to evaluate the accuracy of the ability of CVPT0, ΔCVPT5, ΔCVPT10, ΔCVPT15, and RespCVP to predict fluid responsiveness. The best cutoff values were also calculated via the Youden index method.

The effects of the fluid challenge on hemodynamic variables were assessed via Wilcoxon's rank-sum test or a paired t test, as appropriate. The hemodynamic variables before the fluid challenge in responders and nonresponders were compared via the Mann–Whitney U test or a t test, as appropriate.

The data were analyzed via the ggplot2 package in R 3.4.3 (R Core Team, 2017).

## RESULTS

From May 2016 to September 2017, we evaluated 35 nonconsecutive patients during a fluid challenge. Five of the patients were excluded because of abnormal CVP waveform patterns (2 with regurgitant cv waves, 1 with a whip artifact, and 2 with overdamped-appearing waveforms). The main indications for the fluid challenge were to reduce the use of vasopressors (27 patients, 90%) and hyperlactatemia (12 patients, 40%). The vast majority of the patients (93.3%) were using noradrenaline. Echocardiography was performed in 20 patients and did not reveal features of acute right ventricular failure^(20)^ or valvular lesions. The patient characteristics are presented in table 1.

**Table 1:** Patient characteristics

Patient characteristics			
Age (years)			56.0 ± 14.2
Male sex (%)			18 (60)
BMI (kg/cm²)			27.1 ± 3.7
SAPS 3			72.3 ± 16.0
SOFA score			12.1 ± 2.8
Reason for ICU admission			
		Septic shock	13 (43.3)
		Major abdominal surgery	10 (33.3)
		Major neurosurgery	3 (10.0)
		Coronary artery bypass grafting	2 (6.7)
		Major orthopedic surgery	1 (3.3)
		Neurogenic shock	1 (3.3)
Indication for a fluid challenge*			
		To reduce the vasopressor dose	27(90.0)
		Hyperlactatemia	12 (40.0)
		Tachycardia	4 (13.3)
		Low ScvO_2_	2 (6.7)
		Oliguria	1 (3.3)
Vasoactive drugs			
	Noradrenaline		28 (93.3)
		Median dose (mcg/kg/minute)	0.48 (0.22 - 0.86)
	Adrenaline		10 (33.3)
		Median dose (mcg/kg/minute)	0.22 (0.05 - 0.33)
		Vasopressin	9 (30.0)
		Dobutamine	5 (16.7)
Mean arterial pressure (mmHg)			76.6 ± 9.0
Cardiac index (L/minute/m^2^)			3.45 ± 1.02
Central venous pressure (mmHg)			6.01 ± 3.61
RespCVP (%)			1.8 (-15.27 - 15.84)
Hemoglobin (g/dL)			10.1 ± 3.0
Lactate (mg/dL)			18.2 (14.7 - 48.2)
ScvO_2_ (%)			77.0 (66.5 - 81.2)
PEEP (cmH_2_O)			8.0 (5.0 - 10.0)
Tidal volume (mL)			429 ± 111.7
Cstat (mL/cmH_2_O)			35.0 (27.4 - 46.7)
PaO_2_: FiO_2_			250.8 ± 113.2
Hospital mortality rate			18 (60)

BMI - body mass index; SAPS 3 - Simplified Acute Physiology Score 3; SOFA - Sequential Organ Failure Assessment score; ICU - intensive care unit; ScvO2 - central venous oxygen saturation; RespCVP - cyclic variation in the amplitude of central venous pressure; PEEP - positive end-expiratory pressure; Cstat - respiratory system static compliance; FiO_2_ - fraction of inspired oxygen; PaO_2_ - partial pressure of oxygen.

* a patient could have more than one indication. Variables are expressed as the numbers (%), means (standard deviations) or medians (interquartile ranges: 25^th^ - 75th percentiles).

**Figure 1:**
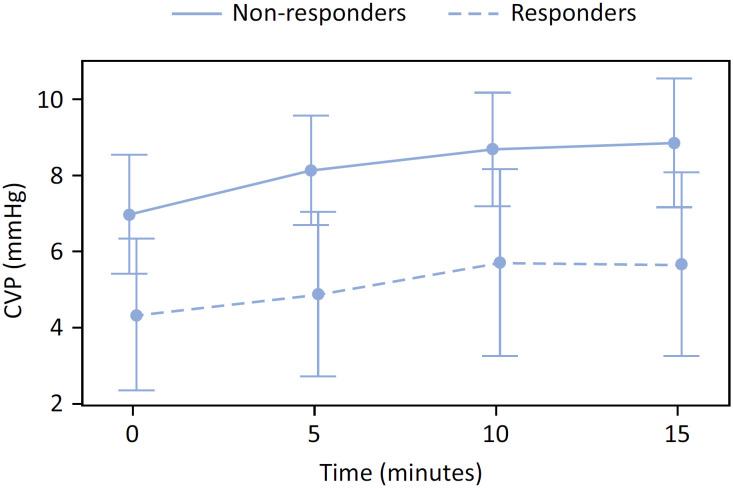
Time course of central venous pressure.

**Figure 2:**
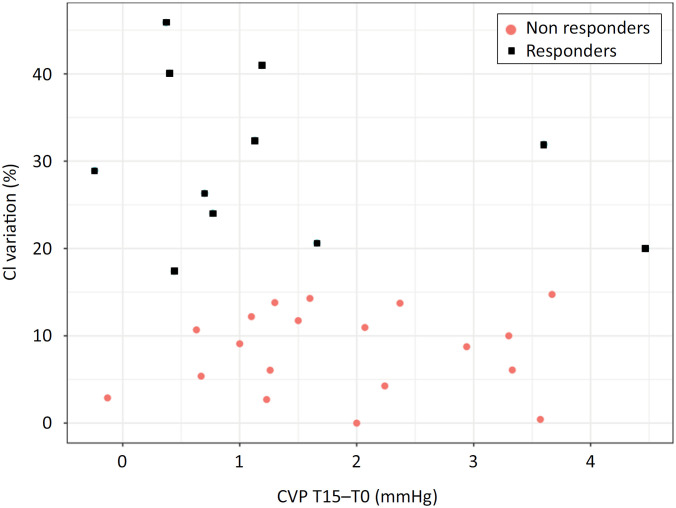
Correlations between changes in central venous pressure and changes in the cardiac index after a fluid challenge.

Among the 30 patients, 11 were responders, and 19 were nonresponders. The mean tidal volume was 6.8 ± 1.4mL/kg of predicted body weight, and the mean static compliance was 35.0 (27.4 - 46.7) mL/cmH_2_O/kg. There were significant increases in CVP over time in both responders and nonresponders (estimate: 0.12, standard error: 0.02, p value < 0.001). However, there were no significant differences between the groups in the mean CVP values (-2.7mmHg, sensitivity [SE]: 1.37, p value = 0.06) or in the mean CVP changes up to 15 minutes (-0.0mmHg, SE: 0.03mmHg, p value = 0.25). The changes in CVP following the fluid challenge are shown in figure 1. The changes in the percentage of the cardiac index did not correlate with the ΔCVPT15 after the fluid challenge (Figure 2).

The central venous pressure tended to be lower in responders (estimate: −2.7, standard error: 1.37, p value: 0.06) and increased during fluid infusion (estimate: 0.12, standard error: 0.02, p value < 0.001). The mean central venous pressure changes up to 15 minutes were similar across the groups at all time points (estimate: −0.0, standard error: 0.03, p value: 0.25).

CVP - central venous pressure.

The changes in the percentage of the cardiac index (cardiac index variation in %) did not correlate with the changes in central venous pressure following a fluid challenge (ΔCVP: T15-T0 in mmHg) (R= −0.25, p value: 0.18).

CI - cardiac index.

The baseline CVP and the changes after the fluid challenge at all three time points did not adequately predict fluid responsiveness. The performance of the ΔCVPT10 when the cutoff was set at 2mmHg, as classically recommended in fluid challenges, was poor, as 74% of the nonresponders had a CVP change < 2mmHg. The RespCVP at baseline also showed a poor performance (Table 2).

**Table 2:** Predictive performance of central venous pressure and cyclic variation in the amplitude of central venous pressure

Variable	AUC	95%CI	Cutoff	Sensitivity (%)	Specificity (%)	PPV (%)	NPV (%)
Baseline CVP	0.70	0.49 - 0.90	6.2	81.8	52.6	50	83.3
ΔCVPT5	0.78	0.57 - 0.99	0.5	81.8	78.9	69.2	88.2
ΔCVPT10	0.63	0.39 - 0.88	0.4	36.4	100.0	100.0	73.1
ΔCVPT15	0.68	0.45 - 0.92	1.2	72.7	73.7	61.5	82.4
ΔCVP	0.70	0.50 - 0.91	-1.9	81.8	57.9	52.9	84.6

AUC - area under the receiver operating characteristic curve; 95%CI - 9%% confidence interval; PPV - positive predictive value; NPV - negative predictive value; CVP - central venous pressure; ΔCVPT5 - difference between the central venous pressure measured at 5 minutes and at baseline; ΔCVPT10 - difference between the central venous pressure measured at 10 minutes and at baseline; ΔCVPT15 - difference between the central venous pressure measured at 15 minutes and at baseline; ΔCVP - cyclic variation in the amplitude of central venous pressure.

The individual changes in CVP (Figure 1S), as well as the hemodynamic variables before and after the fluid challenge (Table 1S), are provided in the Supplementary Material.

## DISCUSSION

The main finding of our study is that the changes in CVP during a fluid challenge, as well as the RespCVP at baseline, have limited value for guiding fluid management in patients under mechanical ventilation.

Fluid infusion is one of the most common interventions in critically ill patients and is a part of the treatment of most forms of shock.^(1,21,22)^ However, repeated fluid infusions can result in fluid overload, which is associated with complications and increased mortality.^(23,24)^ Among patients with inadequate tissue perfusion, identifying those who will increase the stroke volume upon fluid loading (fluid responders) is theoretically a way to avoid the negative effects of excess fluid.^(2,25)^ Central venous pressure and other static parameters have well-known limitations for identifying fluid responders.^(9,26,27)^ Even dynamic parameters, based on heart-lung interactions, have limited applicability in daily practice.^(28,29)^ When the response to fluids is not predictable, a fluid challenge technique is commonly used.^(13,30,31)^ However, an important drawback of a fluid challenge technique is that it is not a test but rather the treatment itself,^(31)^ which means that a patient requiring multiple fluid challenges may receive fluids unnecessarily. Static values of CVP are classically used as a safety limit during a fluid challenge. Pulmonary edema due to congestive heart failure is the most feared complication of fluid infusion.^(13,32)^ However, pulmonary edema depends on the left atrial pressure and pulmonary capillary permeability, and CVP cannot be used to predict it.^(3)^

Another role attributed to CVP during fluid expansion is its use as a dynamic parameter to define when to stop administering fluids, thus avoiding excessive fluid infusion.^(7,13)^ An increase in the mean systemic pressure after fluid infusion is expected to occur in both responders and nonresponders. Previous studies have suggested that in responders, the variation in CVP is smaller than that in nonresponders, resulting in an increase in the venous return gradient.^(33,34)^ However, our study revealed that nonresponders could experience slight changes in CVP during fluid infusion. Recent studies have shown that passive leg raising responders and nonresponders do not exhibit significant differences in CVP variation induced by the maneuver,^(35,36)^ which aligns with our findings. Thus, the suggestion to rely solely on the CVP as a stopping criterion may be misleading.^(17)^ Some studies have already demonstrated a poor correlation between changes in CVP and those in the stroke index/cardiac index.^(6,37)^ Therefore, our findings suggesting that changes in CVP have limited value in predicting fluid responsiveness are, to some extent, not novel.

The RespCVP indicator also performed poorly in our study. In 30 postoperative cardiac surgery patients, Westphal et al. demonstrated that changes in the CVP amplitude induced by mechanical ventilation adequately discriminated potential fluid responders (patients who had a PPV ≥ 13%) from potential fluid nonresponders (patients who had a PPV < 13%), suggesting that it may be used as a dynamic marker of fluid responsiveness.^(18)^ We could not reproduce these results. Other attempts to use the concept of CVP curve variation to predict fluid responsiveness were previously proposed in patients with spontaneous respiratory movements but have not been reproduced.^(38,39)^

Importantly, our findings reinforce the limitations of CVP in guiding fluid therapy.^(6)^ However, we agree that CVP can provide important insights into the cardiovascular status and the likelihood of diagnosis and should not be ruled out in decision-making regarding the infusion of fluids.^(7,40)^ For example, although some patients may respond to fluids at high levels of CVP, the response is less likely, and the risk of systemic congestion increases.^(7,10,40,41)^ Furthermore, monitoring changes in CVP can be informative during fluid administration if analyzed in conjunction with changes in the cardiac output. A significant increase in CVP, with little to no change in the cardiac output, suggests poor tolerance to fluids. On the other hand, a minimal change in CVP, paired with an increase in the cardiac output, indicates fluid responsiveness.^(7)^

The ΔCVPT15 was less than 2mmHg in most responders, whereas its values were equally distributed from below 0 to 3.5mmHg in the nonresponders. We believe that these findings could be due to chance and attributable to the small sample size. This does not invalidate the findings, as there is a significant overlap in values.

Our study has several strengths. First, we measured both the static CVP and its dynamic changes, including the RespCVP, which allowed a complete assessment of this hemodynamic variable. Second, we carefully assessed CVP and infused fluids in a controlled setting to prevent the influence of factors other than infusion itself that could lead to changes in CVP. Third, we determined CVP within short periods of time, allowing the proper assessment of acute hemodynamic changes, which could allow timely assessment of fluid responsiveness early in the course of the fluid challenge. Fourth, our patients were severely ill, which allowed a pragmatic evaluation of the utility of CVP.

However, our study has several limitations. First, our sample size was small. As studies suggest that changes in CVP could be useful for predicting the response to additional fluid infusion,^(33,42)^ a larger sample size would strengthen our findings. Second, the amount of the infused fluid might not have been enough to impact the cardiac output. If the cardiac output does not increase with a volume bolus, this could be due to an insufficient volume that was given to challenge the right cardiac function curve.^(40)^ We believe that the amount of fluid (500mL of crystalloids over 15 minutes) was adequate, despite minimal changes in CVP, because the variation in CVP following the fluid challenge was similar in the responders and nonresponders. Moreover, there is evidence suggesting that volumes between 320 and 510mL may be sufficient for an effective fluid challenge.^(43)^ Third, we did not perform echocardiography in all patients; however, among the 20 patients who were analyzed, right ventricular function was preserved. We cannot rule out that our findings would have been different in the presence of right ventricular dysfunction, as an increase in the right ventricular volume would theoretically result in a marked increase in CVP in nonresponsive patients. Fourth, three different methods were used to obtain the cardiac index, including arterial pulse contour analysis, which require frequent recalibration to increase accuracy.^(44)^ Fifth, the heterogeneity of the population should also be noted among the limitations of the study. Finally, our inclusion of patients was not consecutive, and it took a long time to include all patients.

## CONCLUSION

In critically ill adult patients on mechanical ventilation, dynamic changes in central venous pressure have limited value with respect to guiding fluid management. Central venous pressure changes up to 15 minutes following fluid infusion, and the RespCVP at baseline should not be used as a marker of the response to fluids.
