# Supplementary material for: Changes in central venous pressure during a fluid challenge have limited value for guiding fluid therapy
Source: Crit Care Sci. 2024 Nov 14;36:e20240073en. doi: 10.62675/2965-2774.20240073-en (PMC11634234; doi:10.62675/2965-2774.20240073-en)
Supplement: Supplementary file 1 [file 2965-2774-ccsci-36-e20240073en-Suppl01.pdf]

Critical Care Science
https://doi.org/10.62675/2965-2774.20240073-en

SUPPLEMENTARY MATERIAL

Changes in central venous pressure during a fluid challenge have limited value for guiding fluid therapy

Priscilla Souza de Oliveira[1], Fernando José da Silva Ramos[1], Daniere Yurie Vieira Tomotani[1], Flávia Ribeiro Machado[1], Flávio Geraldo Rezende de Freitas[1]

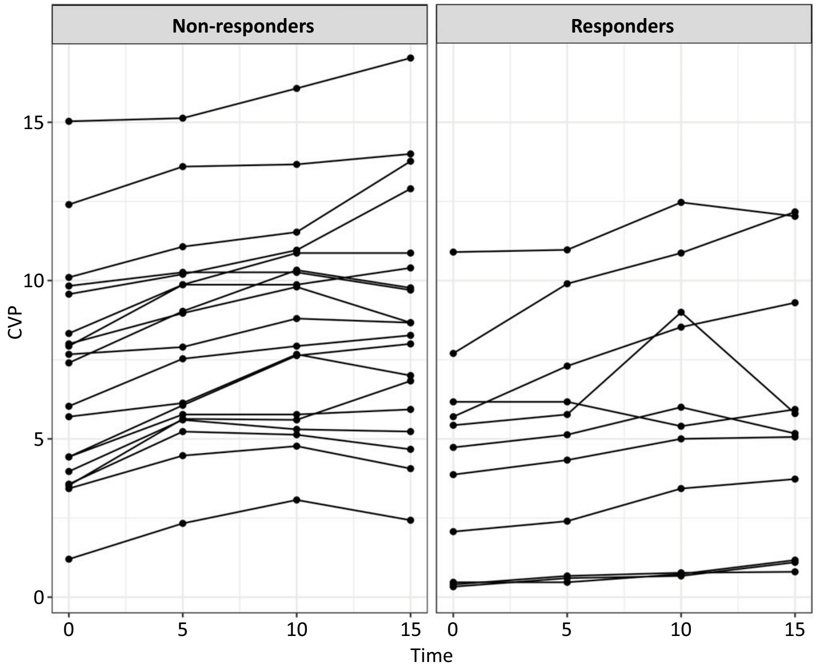

Figure 1S - Individual time-course changes of central venous pressure.
CVP - central venous pressure.

This is an open access article under the CC BY license (https://creativecommons.org/licenses/by/4.0/).

Table 1S - Hemodynamic variables before and after fluid

Variable	Responders (n = 11)		Non-responders (n = 19)	
	Before fluid	After fluid	Before fluid	After fluid
CVP (mmHg)	4.3 ± 3.4	4.9 ± 3.6*	7.0 ± 3.5	8.1 ± 3.2*
RespCVP (%)	6.0 (-1.9 - 20.1)	15.6 (-8,8 - 23.5)	-5.2 (-17.8 - 6.7)	1.2 (-13.7 - 13.3)
HR (beats.minute^{-1})	119.5 ± 31.4	112.9 ± 31.2*	101.2 ± 22.6	97.5 ± 19.9*
MAP (mmHg)	75.6 ± 9.1	89.3 ± 7.3*	77.2 ± 9.1	87.8 ± 13.1*
Hemoglobin (g/dL)	10.7 ± 2.7	10.3 ± 2.7*	10.2 ± 2.6	10.2 ± 2.4
Lactate (mg/dL)	19 (10 - 59)	22 (10 - 50)	18 (15 - 48)	20 (16 - 42)
$ScvO_2$ (%)	78 (67 - 81)	82 (72 - 85)	76 (63 - 81)	76 (65 - 84)
CI (L/minute/m^2)	3.45 ± 0.9	4.48 ± 1.3*	3.45 ± 1.0	3.7 ±1.1*

CVP - central venous pressure; RespCVP - cyclic variation in amplitude of central venous pressure; HR - heart rate; MAP - mean arterial pressure - $ScvO_2$ - central venous oxygen saturation; CI - cardiac index. * $p < 0.05$ *versus* baseline. There is no difference between responders and non-responders at baseline. Data are presented as the means (standard deviation) or medians (interquartile range: 25 - 75th percentile).
