# Supplementary material for: Changes in central venous pressure during a fluid challenge have limited value for guiding fluid therapy
Source: Crit Care Sci. 2024 Nov 14;36:e20240073en. doi: 10.62675/2965-2774.20240073-en (PMC11634234; doi:10.62675/2965-2774.20240073-en)
Supplement: Translation

# Translation: Variações na pressão venosa central durante um desafio volêmico têm valor limitado para orientar a terapia com fluidos

**DOI:** 10.62675/2965-2774.20240073-pt

**Authors:** Priscilla Souza de Oliveira, Fernando José da Silva Ramos, Daniere Yurie Vieira Tomotani, Flávia Ribeiro Machado, Flávio Geraldo Rezende de Freitas

**Affiliations:** 1 São Paulo SP Brasil Departamento de Anestesiologia, Dor e Terapia Intensiva, Escola Paulista de Medicina, Universidade Federal de São Paulo - São Paulo (SP), Brasil.

**Keywords:** Pressão venosa central, Cuidados críticos, Hidratação, Respiração artificial, Débito cardíaco

## Abstract

**Objetivo:**

Avaliar se as variações na pressão venosa central durante a expansão volêmica e a variação respiratória cíclica basal na curva de amplitude da pressão venosa central poderiam ser usadas para discriminar entre pacientes responsivos e não responsivos a fluidos.

**Métodos:**

Trata-se de um estudo observacional prospectivo que incluiu pacientes adultos em estado crítico que foram submetidos à expansão volêmica na forma de infusão volêmica ou desafio volêmico de cristaloides. Todos os pacientes estavam em ventilação mecânica e devidamente sedados. Determinamos a pressão venosa central basal (PVCT0) e as variações em 5 (ΔPVCT5), 10 (ΔPVCT10) e 15 (ΔPVCT15) minutos durante a infusão volêmica. Também medimos a curva de amplitude da PVCT0. Definiu-se a responsividade a fluidos como um aumento do índice cardíaco de ≥ 15%.

**Resultados:**

O estudo incluiu 30 pacientes (11 responsivos e 19 não responsivos). A PVCT0 e as variações após um desafio volêmico em todos os três momentos não previram adequadamente a capacidade de resposta a fluidos, conforme determinado por seus valores de área sob a curva (PVCT0: 0,70, IC95% 0,49 - 0,90; ΔPVCT5: 0,78, IC95% 0,57 - 0,99; ΔPVCT10: 0,63, IC95% 0,39 - 0,88; ΔPVCT15: 0,68; IC95% 0,45 - 0,92). A curva de amplitude da PVCT0 também teve um desempenho insatisfatório (área sob a curva: 0,70; IC95% 0,50 - 0,91).

**Conclusão:**

As variações na pressão venosa central têm valor limitado na previsão da responsividade a fluidos.

## INTRODUÇÃO

Os fluidos intravenosos são geralmente considerados como o primeiro passo na ressuscitação de pacientes críticos com sinais de má perfusão tecidual.^(1,2)^ A justificativa para a infusão volêmica é aumentar o retorno venoso, o que leva a um aumento no débito cardíaco e melhora na perfusão dos órgãos.^(3)^ No entanto, estudos clínicos em populações heterogêneas de pacientes críticos demonstraram que o débito cardíaco aumenta em apenas aproximadamente 50% dos pacientes hemodinamicamente instáveis após a infusão de fluidos.^(4,5)^

A pressão venosa central (PVC) é uma das variáveis hemodinâmicas mais tradicionais usadas para prever a responsividade volêmica. Sua ampla utilização baseia-se no dogma de que a PVC reflete o volume intravascular.^(6)^ Entretanto, a PVC representa uma complexa interação de fatores, incluindo a complacência cardíaca, a função ventricular direita, a pressão intratorácica, o tônus vascular e o volume circulante. Consequentemente, as mudanças na PVC podem não refletir apenas alterações na pré-carga cardíaca. Inúmeros estudos demonstraram de forma contundente que os valores absolutos da PVC não refletem com precisão o volume intravascular e são ineficazes para distinguir os responsivos dos não responsivos.^(2,3,6-11)^ Apesar das evidências de longa data que sugerem que a PVC não é adequada para guiar a terapia com fluidos, em 2015 um amplo estudo observacional descreveu como os médicos conduzem os desafios volêmicos na terapia intensiva e revelou que a PVC ainda era a variável preferida para prever a responsividade a fluidos.^(12)^

Ao contrário dos valores estáticos da PVC, há uma crença geral de que as variações dinâmicas da PVC podem ser úteis, especialmente quando não há acesso a sistemas de monitoramento mais avançados.^(13-15)^ A estratégia mais comumente utilizada fundamenta-se nas variações da PVC no decorrer de um desafio volêmico para orientar as infusões volêmicas subsequentes, conforme proposto por Weil e Henning na regra "5-2".^(16)^ De acordo com essa estratégia, variações mínimas da PVC, como aumento abaixo de 2mmHg, podem ser indicativas de responsividade a fluidos, levando à infusão de mais volume.^(13,16,17)^ Outro método interessante é a variação cíclica na amplitude da PVC em pacientes em ventilação mecânica, proposto por Westphal et al.^(18)^ Esse índice foi relatado anteriormente como estando em conformidade com a variação na pressão de pulso, sugerindo que pode ser usado como um marcador dinâmico de responsividade a fluidos.

Assim, levantamos a hipótese de que as variações na PVC durante a expansão volêmica em pacientes críticos em ventilação mecânica poderiam ajudar a identificar o estado de responsividade a fluidos. Nosso objetivo foi avaliar se as variações na pressão venosa central durante a expansão volêmica e a variação respiratória cíclica basal na curva de amplitude da pressão venosa central poderiam ser usadas para discriminar entre pacientes responsivos e não responsivos a fluidos.

## MÉTODOS

Trata-se de um estudo observacional prospectivo realizado em duas unidades de terapia intensiva mistas (35 leitos e 16 leitos) em dois hospitais universitários. O estudo foi aprovado pelos Comitês de Ética de ambas as instituições. Obteve-se o Termo de Consentimento Livre e Esclarecido dos representantes legais dos pacientes.

Incluímos pacientes adultos que receberam expansão volêmica na forma de infusão volêmica ou desafio volêmico com cristaloides (Ringer com lactato ou cloreto de sódio a 0,9%; 500mL infundidos durante 15 minutos), conforme prescrição do médico assistente. Somente pacientes em sedação contínua, totalmente adaptados ao ventilador e monitorados com um cateter arterial e um dispositivo para medir o débito cardíaco foram considerados aptos para inclusão neste estudo. Excluímos pacientes com sangramento ativo (suspeito ou confirmado), necessidade urgente de reposição volêmica, arritmias, derivações intracardíacas conhecidas, doença da válvula tricúspide e rastreamento de baixa qualidade da PVC.

Na medição da pressão, usamos um monitor multiparamétrico de beira de leito (DX 2020, Philips-Dixtal, São Paulo, Brasil). Determinamos a PVC basal e aos 5, 10 e 15 minutos de infusão volêmica. Todas as medições de PVC foram realizadas em triplicata e calcularam-se as médias. Medimos a PVC com um cateter venoso central em todos os pacientes, incluindo aqueles que foram monitorados com um cateter de artéria pulmonar. Determinamos a posição da ponta do cateter venoso central por meio de uma radiografia de tórax à beira do leito. Para determinar a PVC, congelamos a tela do monitor com sinais simultâneos de rastreamento da PVC, eletrocardiograma e capnografia. Posicionamos a linha do cursor na parte inferior da onda a no final da expiração.^(19)^ As variações na PVC durante um desafio volêmico foram definidas da seguinte forma: ΔPVCT5, ΔPVCT10 e ΔPVCT15 corresponderam às diferenças entre os valores de PVC medidos em 5, 10 e 15 minutos e o valor basal, respectivamente. A amplitude da PVC foi calculada da parte superior para a parte inferior do traçado da onda a, tanto na fase inspiratória (PVCins) quanto na fase expiratória (PVCexp). Calculamos a curva de amplitude da PVC com a seguinte fórmula: ΔPVC(%)=[( PVCexp− PVCins)/ PVCexp]×100.^(18)^

Inicialmente e imediatamente após o desafio volêmico, registrou-se um conjunto de variáveis hemodinâmicas, inclusive a frequência cardíaca, a pressão arterial média e o índice cardíaco. Para garantir a precisão das medições da pressão intravascular, observamos cuidadosamente a permeabilidade da linha, o nivelamento do transdutor (linha axilar média no quarto interespaço), a zeragem à pressão atmosférica e a qualidade do sinal (teste de onda quadrada). Todos os demais tratamentos, incluindo medicamentos sedativos e vasoativos, não foram modificados durante o estudo.

Medimos o índice cardíaco com o sistema PiCCO (Pulsion Medical Systems SE, Munique, Alemanha), o sistema VolumeView/EV1000 (Edwards Lifesciences, Irvine, Califórnia, Estados Unidos) ou o débito cardíaco sem continuo com um cateter de artéria pulmonar (Vigilance, Edwards Lifesciences, Irvine, Califórnia, Estados Unidos). Para os sistemas PiCCO e EV1000, o índice cardíaco foi determinado mediante análise do contorno do pulso. De acordo com o protocolo local, a calibração foi repetida a cada 8 horas ou após alteração importante na condição clínica do paciente. Considerou-se, para o cateter de artéria pulmonar, o valor médio de cinco medições consecutivas da tela do modo STAT do monitor Vigilance^®^ (Edwards Lifesciences, Irvine, Califórnia, Estados Unidos) após a conclusão do desafio volêmico. Os pacientes foram classificados como responsivos se o desafio volêmico induzisse a um aumento no índice cardíaco de ≥ 15%.

Também obtivemos variáveis clínicas e epidemiológicas em prontuários médicos. Calculamos o escore *Sequential Organ Failure Assessment* (SOFA) no dia do protocolo e o *Simplified Acute Physiology Score* (SAPS) 3 na admissão à unidade de terapia intensiva (UTI).

### Análise estatística

Para calcular o tamanho da amostra, consideramos uma área sob a curva Característica de Operação do Receptor (ROC - *receiver operating characteristic*) de 0,85 ou mais para a variável ΔPVCT15 para prever a responsividade a fluidos, considerando um nível alfa de 0,05, um nível beta de 0,20 e uma proporção de responsivos e não responsivos de 1:1, sendo necessário um mínimo de nove pacientes por grupo.

Os dados são expressos como número (porcentagem), média ± desvio-padrão ou mediana (intervalo interquartil), conforme o caso. Os dados contínuos foram testados quanto à normalidade da distribuição por meio do teste de Shapiro-Wilk. Para comparar os valores de PVC entre responsivos e não responsivos ao longo do tempo, usamos um modelo misto com efeitos aleatórios individuais. Testamos a correlação entre as variações no índice cardíaco (em porcentagem) e as variações na PVC após um desafio volêmico (ΔPVCT15) pelo método de classificação de Spearman.

As curvas ROC foram calculadas para avaliar a precisão da capacidade da PVCT0, ΔPVCT5, ΔPVCT10, ΔPVCT15 e amplitude da PVC de prever a responsividade volêmica. Os melhores valores de corte também foram calculados pelo método do índice de Youden.

Os efeitos do desafio volêmico nas variáveis hemodinâmicas foram avaliados por meio do teste de soma de classificação de Wilcoxon ou de um teste *t* pareado, conforme o caso. As variáveis hemodinâmicas antes do desafio volêmico em responsivos e não responsivos foram comparadas com o teste U de Mann-Whitney ou um teste *t*, conforme o caso.

Os dados foram analisados por meio do pacote ggplot2 no R 3.4.3 (R Core Team, 2017).

## RESULTADOS

De maio de 2016 a setembro de 2017, avaliamos 35 pacientes não consecutivos durante um desafio volêmico. Foram excluídos cinco pacientes devido a padrões anormais de forma de onda de PVC (dois com ondas cv regurgitantes, um com um artefato de chicote e dois com formas de onda com aparência de superamortecimento). As principais indicações do desafio volêmico foram a redução do uso de vasopressores (27 pacientes, 90%) e a hiperlactatemia (12 pacientes, 40%). A maioria dos pacientes (93,3%) usava noradrenalina. O ecocardiograma foi realizado em 20 pacientes e não revelou características de insuficiência ventricular direita aguda^(20)^ ou lesões valvares. A tabela 1 apresenta as características dos pacientes.

**Tabela 1:** Características dos pacientes

Características dos pacientes			
Idade (anos)			56,0 ± 14,2
Sexo masculino (%)			18 (60)
IMC (kg/cm²)			27,1 ± 3,7
SAPS 3			72,3 ± 16,0
SOFA			12,1 ± 2,8
Motivo da admissão na UTI			
	Choque séptico		13 (43,3)
	Cirurgia abdominal de grande porte		10 (33,3)
	Cirurgia neurológica de grande porte		3 (10,0)
	Cirurgia de revascularização do miocárdio		2 (6,7)
	Cirurgia ortopédica de grande porte		1 (3,3)
	Choque neurogênico		1 (3,3)
Indicação para desafio volêmico*			
	Diminuir a dose de vasopressor		27(90,0)
	Hiperlactatemia		12 (40,0)
	Taquicardia		4 (13,3)
	SvcO_2_ baixa		2 (6,7)
	Oligúria		1 (3,3)
Drogas vasoativas			
	Noradrenalina		28 (93,3)
		Dose mediana (mcg/kg/minuto)	0,48 (0,22 - 0,86)
	Adrenalina		10 (33,3)
		Dose mediana (mcg/kg/minuto)	0,22 (0,05 - 0,33)
	Vasopressina		9 (30,0)
	Dobutamina		5 (16,7)
Pressão arterial média (mmHg)			76,6 ± 9,0
Índice cardíaco (L/minuto/m_2_)			3,45 ± 1,02
Pressão venosa central (mmHg)			6,01 ± 3,61
Variação cíclica na amplitude da PVC (%)			1,8 (-15,27 - 15,84)
Hemoglobina (g/dL)			10,1 ± 3,0
Lactato (mg/dL)			18,2 (14,7 - 48,2)
SvcO_2_ (%)			77,0 (66,5 - 81,2)
PEEP (cmH_2_O)			8,0 (5,0 - 10,0)
Volume corrente (mL)			429 ± 111,7
Cstat (mL/cmH_2_O)			35,0 (27,4 - 46,7)
PaO_2_/FiO_2_			250,8 ± 113,2
Taxa de mortalidade hospitalar			18 (60)

IMC - índice de massa corporal; SAPS 3 - *Simplified Acute Physiology Score 3*; SOFA - *Sequential Organ Failure Assessment*; UTI - unidade de terapia intensiva; SvcO_2_ - saturação venosa central de oxigênio; PVC - pressão venosa central; PEEP - pressão positiva expiratória final; Cstat - complacência estática do sistema respiratório; PaO_2_/FiO_2_ – relação entre pressão parcial de oxigênio e fração inspirada de oxigênio.

* Um paciente pode ter mais de uma indicação. Resultados expressos como média ± desvio-padrão, n (%) ou mediana (intervalo interquartil: percentis 25 a 75).

Dos 30 pacientes, 11 responderam e 19 não responderam. O volume corrente médio foi de 6,8 ± 1,4mL/kg de peso corporal previsto e a complacência estática média foi de 35,0 (27,4 - 46,7) mL/cmH_2_O/kg. Observaram-se aumentos significativos na PVC ao longo do tempo, tanto nos responsivos quanto nos não responsivos (estimativa: 0,12, erro-padrão: 0,02, valor de p < 0,001). Entretanto, não houve diferenças significativas nos valores médios de PVC entre os grupos (-2,7mmHg, sensibilidade de 1,37, valor de p = 0,06) ou nas variações médias de PVC até 15 minutos (-0,0mmHg, sensibilidade de 0,03mmHg, valor de p = 0,25). A figura 1 apresenta as variações na PVC após o desafio volêmico. As variações percentuais do índice cardíaco não se correlacionaram com a ΔPVCT15 após o desafio volêmico (Figura 2).

**Figura 1:** Curso temporal da pressão venosa central.

A pressão venosa central tendeu a ser menor nos responsivos (estimativa: −2,7, erro-padrão: 1,37, valor de p: 0,06) e aumentou durante a infusão de fluidos (estimativa: 0,12, erro-padrão: 0,02, valor de p < 0,001). As variações médias da pressão venosa central até 15 minutos foram semelhantes em todos os grupos e em todos os momentos (estimativa: −0,0, erro-padrão: 0,03, valor de p: 0,25).

PVC - pressão venosa central.

**Figura 2:** Correlações entre as variações na pressão venosa central e as variações no índice cardíaco após um desafio de fluidos.

As variações na porcentagem do índice cardíaco (variação do índice cardíaco em %) não se correlacionaram com as variações na pressão venosa central após um desafio de fluidos (ΔPVC: T15-T0, em mmHg) (R= −0,25, valor de p: 0,18).

IC - índice cardíaco; PVC - pressão venosa central.

A PVC basal e as variações após o desafio volêmico em todos os três momentos não previram adequadamente a responsividade a fluidos. O desempenho da ΔPVCT10 foi insatisfatório quando o ponto de corte foi definido em 2mmHg, como classicamente recomendado em desafios volêmicos, pois 74% dos não responsivos tiveram uma variação na PVC < 2mmHg. A amplitude da PVC basal também apresentou um desempenho insatisfatório (Tabela 2).

**Tabela 2:** Desempenho preditivo da pressão venosa central e variação cíclica na amplitude da pressão venosa central

Variável	AUC	IC95%	Ponto de corte	Sensibilidade (%)	Especificidade a(%)	VPP (%)	VPN (%)
PVC basal	0,70	0,49 - 0,90	6,2	81,8	52,6	50	83,3
ΔPVCT5	0,78	0,57 - 0,99	0,5	81,8	78,9	69,2	88,2
ΔPVCT10	0,63	0,39 - 0,88	0,4	36,4	100,0	100,0	73,1
ΔPVCT15	0,68	0,45 - 0,92	1,2	72,7	73,7	61,5	82,4
ΔPVC	0,70	0,50 - 0,91	-1,9	81,8	57,9	52,9	84,6

AUC - área sob a curva; IC95% - intervalo de confiança de 95%; VPP - valor preditivo positivo; VPN - valor preditivo negativo; PVC - pressão venosa central; ΔPVCT5 - diferença entre a pressão venosa central medida em 5 minutos e a PVC basal; ΔPVCT10 - diferença entre a pressão venosa central medida em 10 minutos e a PVC basal; ΔPVCT15 - diferença entre a pressão venosa central medida em 15 minutos e a PVC basal; ΔPVC - variação cíclica na amplitude da pressão venosa central.

As variações individuais na PVC (Figura 1S), bem como as variáveis hemodinâmicas antes e depois do desafio volêmico (Tabela 1S), encontram-se no Material Suplementar.

## DISCUSSÃO

O principal achado de nosso estudo é que as variações na PVC durante um desafio volêmico, bem como a amplitude da PVC basal, têm valor limitado para orientar o manejo de fluidos em pacientes em ventilação mecânica.

A infusão volêmica é uma das intervenções mais comuns em pacientes críticos e faz parte do tratamento da maioria das formas de choque.^(1,21,22)^ No entanto, repetidas infusões volêmicas podem resultar em sobrecarga de volume, que está associada a complicações e ao aumento da mortalidade.^(23,24)^ A identificação de pacientes com perfusão tecidual inadequada que aumentarão o volume sistólico após a carga de volume (responsivos a fluidos) é, teoricamente, uma forma de evitar os efeitos negativos do excesso de fluidos.^(2,25)^ A PVC e outros parâmetros estáticos têm limitações bem conhecidas para identificar os responsivos a fluidos.^(9,26,27)^ Até mesmo os parâmetros dinâmicos, fundamentados nas interações coração-pulmão, têm aplicabilidade limitada na prática diária.^(28,29)^ Quando a responsividade a fluidos não é previsível, utiliza-se com frequência uma técnica de desafio de fluidos.^(13,30,31)^ No entanto, uma desvantagem importante da técnica de desafio de fluidos é que ela não é um teste, mas sim o próprio tratamento,^(31)^ o que significa que um paciente que precisa de vários desafios de fluidos pode receber fluidos desnecessariamente. Os valores estáticos da PVC são usados classicamente como um limite de segurança durante um desafio de fluidos. O edema pulmonar devido à insuficiência cardíaca congestiva é a complicação mais temida da infusão volêmica.^(13,32)^ No entanto, o edema pulmonar depende da pressão atrial esquerda e da permeabilidade capilar pulmonar, e a PVC não pode ser usada para prever isso.^(3)^

Outro papel atribuído à PVC durante a expansão volêmica é seu uso como um parâmetro dinâmico para definir quando interromper a infusão de volume, evitando assim a infusão volêmica excessiva.^(7,13)^ Espera-se que ocorra um aumento na pressão sistêmica média após a infusão volêmica tanto em responsivos quanto em não responsivos. Estudos anteriores sugeriram que, nos responsivos, a variação na PVC é menor do que nos não responsivos, resultando em um aumento no gradiente de retorno venoso.^(33,34)^ No entanto, nosso estudo revelou que os não responsivos poderiam experimentar pequenas variações na PVC durante a infusão volêmica. Estudos recentes mostraram que os responsivos e os não responsivos à elevação passiva das pernas não apresentam diferenças significativas na variação na PVC induzida pela manobra,^(35,36)^ o que coincide com nossos achados. Assim, pode ser enganosa a sugestão de confiar apenas na PVC como critério de interromper.^(17)^ Alguns estudos já demonstraram uma correlação insatisfatória entre as variações na PVC e as variações no índice de AVC/índice cardíaco.^(6,37)^ Portanto, nossos achados sugestivos de que as variações na PVC têm valor limitado na previsão da responsividade a fluidos não são, até certo ponto, novos.

O indicador amplitude da PVC também teve desempenho insatisfatório em nosso estudo. Westphal et al. demonstraram, em 30 pacientes pós-operatórios de cirurgia cardíaca, que as variações na amplitude da PVC induzidas pela ventilação mecânica discriminaram adequadamente os possíveis responsivos a volume (pacientes com variação de pressão de pulso [VPP] ≥ 13%) dos possíveis não responsivos a volume (pacientes com VPP < 13%), sugerindo que ela pode ser aplicada como um marcador dinâmico de responsividade a fluidos.^(18)^ Não conseguimos reproduzir esses resultados. Outras tentativas de usar o conceito de variação da curva na PVC para prever a responsividade a fluidos foram propostas anteriormente em pacientes com movimentos respiratórios espontâneos, mas não foram reproduzidas.^(38,39)^

Vale ressaltar que nossos achados reforçam as limitações da PVC na orientação da hidratação.^(6)^ No entanto, concordamos que a PVC pode fornecer informações importantes do estado cardiovascular e da probabilidade de diagnóstico e não deve ser descartada na tomada de decisões quanto à infusão volêmica.^(7,40)^ Por exemplo, embora alguns pacientes possam responder a volume em níveis altos de PVC, a resposta é menos provável, e o risco de congestão sistêmica aumenta.^(7,10,40,41)^ Além disso, o monitoramento de variações na PVC pode ser informativo durante a administração de volume se analisado em conjunto com variações no débito cardíaco. Um aumento significativo na PVC, com pouca ou nenhuma variação no débito cardíaco, sugere baixa tolerância a volume. Por outro lado, uma mínima variação na PVC, associada a um aumento no débito cardíaco, indica capacidade de responsividade volêmica.^(7)^

A ΔPVCT15 foi inferior a 2mmHg na maioria dos responsivos, enquanto seus valores foram igualmente distribuídos de abaixo de zero a 3,5mmHg nos não responsivos. Acreditamos que esses achados podem se dever ao acaso e ser atribuídos ao tamanho reduzido da amostra. Isso não invalida os resultados, pois há uma sobreposição significativa de valores.

Nosso estudo tem vários pontos fortes. Primeiro, medimos a PVC estática e suas variações dinâmicas, incluindo a amplitude da PVC, o que permitiu uma avaliação completa dessa variável hemodinâmica. Em segundo lugar, avaliamos cuidadosamente a PVC e o volume infundido em um ambiente controlado para evitar a influência de outros fatores, além da própria infusão, que poderiam levar a variações na PVC. Em terceiro lugar, determinamos a PVC em curtos períodos de tempo, permitindo a avaliação adequada das variações hemodinâmicas agudas, o que poderia permitir a avaliação oportuna da responsividade volêmica durante o desafio volêmico. Quarto, nossos pacientes estavam em estado crítico, o que permitiu uma avaliação pragmática da utilidade da PVC.

No entanto, nosso estudo tem várias limitações. Primeiro, o tamanho da nossa amostra foi reduzido. Como os estudos sugerem que as variações na PVC podem ser úteis para prever a resposta à infusão de mais volume,^(33,42)^ um tamanho de amostra maior fortaleceria nossos achados. Em segundo lugar, a quantidade de volume infundido pode não ter sido suficiente para afetar o débito cardíaco. Se o débito cardíaco não aumentar com uma infusão de volume, isso pode ser devido a um volume insuficiente que foi infundido para testar a curva de função cardíaca direita.^(40)^ Acreditamos que a quantidade de volume (500mL de cristaloides durante 15 minutos) foi adequada, apesar das variações mínimas na PVC, porque a variação na PVC após o desafio volêmico foi semelhante nos responsivos e não responsivos. Além disso, há evidências sugestivas de que volumes entre 320 e 510mL podem ser suficientes para um desafio volêmico eficaz.^(43)^ Em terceiro lugar, não realizamos ecocardiografia em todos os pacientes; no entanto, nos 20 pacientes analisados, a função ventricular direita estava preservada. Não podemos descartar que nossos achados teriam sido diferentes na presença de disfunção do ventrículo direito, pois um aumento no volume do ventrículo direito teoricamente resultaria em um aumento acentuado da PVC em pacientes não responsivos. Em quarto lugar, foram usados três métodos diferentes para obter o índice cardíaco, incluindo a análise do contorno do pulso arterial, que exige recalibração frequente para aumentar a precisão.^(44)^ Em quinto lugar, a heterogeneidade da população também deve ser observada entre as limitações do estudo. Por fim, nossa inclusão de pacientes não foi consecutiva, e levou muito tempo para incluir todos os pacientes.

## CONCLUSÃO

Em pacientes adultos em estado crítico com ventilação mecânica, as variações dinâmicas na pressão venosa central têm valor limitado no que diz respeito à orientação do manejo de fluidos. A pressão venosa central varia até 15 minutos após a infusão volêmica, e a amplitude da pressão venosa central basal não deve ser usada como um marcador da responsividade a fluidos.
